# Phytotherapy of polycystic ovary syndrome: A review

**DOI:** 10.18502/ijrm.v20i1.10404

**Published:** 2022-02-18

**Authors:** Farahnaz Azin, Homayoun Khazali

**Affiliations:** Department of Animal Science and Biotechnology, Faculty of Life Sciences and Biotechnology, Shahid Beheshti University, Tehran, Iran.

**Keywords:** Polycystic ovary syndrome, Endocrine disorders, Phytotherapy.

## Abstract

**Background:**

Polycystic ovary syndrome (PCOS) is a complex heterogeneous disease with various symptoms, which can affect females of reproductive age. Endocrine and metabolic abnormalities such as infertility, being overweight or obese, type 2 diabetes, hyperandrogenism and increased luteinizing hormone (LH) are common in women with PCOS.

**Objective:**

This review aimed to assess the efficacy of non-chemical and herbal substances for PCOS recovery.

**Materials and Methods:**

The keywords “non-chemical treatment”, “herbal treatment”, “polycystic ovary syndrome” and “PCOS” were used to search for articles in the electronic databases PubMed/MEDLINE, Web of Science, Scopus, and Reaxys, published from January 2009 to December 2019.

**Results:**

34 relevant studies were found and were briefly described in this review. The most effective herbal treatments in animal models of PCOS were used to restore abnormality in serum sex steroid profile, LH: follicle stimulating hormone ratio, steroidogenic enzymes, cardiovascular parameters, lipid profile, and glucose and estrous cycles. In PCOS patients, positive effects on PCOS due to reductions in testosterone, estrogen, LH, LH: follicle stimulating hormone ratio, and insulin levels were observed.

**Conclusion:**

The results of this reviewrevealed the variability and efficacy of phytotherapy and non-chemical treatments associated with PCOS disease. These findings may help future studies on the etiology and treatment of this syndrome.

## 1. Introduction

Polycystic ovary syndrome (PCOS) is a heterogeneous endocrine disease with various symptoms, which affects 5-10% of females of reproductive age (1, 2). The main clinical features of PCOS are being overweight, hyperandrogenaemia, polycystic ovarian morphology and hyperinsulinemia (3). Although the etiology of PCOS is not completely understood yet, scientific studies suggest that uncontrolled steroidogenesis may be the primary feature in this syndrome (4). PCOS, as a complex heterogeneous disease, can be associated with various genetic, metabolic, endocrine and environmental abnormalities such as being overweight or obese, type 2 diabetes, impaired glucose tolerance, insulin resistance, hyperandrogenism and increased luteinizing hormone (LH) (5, 6).

Increases in oxidative stress levels and inflammatory markers, LH and androgens, and a significant reduction in follicle stimulating hormone (FSH) and estrogen have been reported in patients with PCOS and also in animal models of PCOS (7, 8). It has been shown that non-chemical and herbal ingredients can have considerable effects on recovery and improvement of some abnormalities and disorders in PCOS patients, and many studies have been conducted on their effects on the hormonal and metabolic factors and hypothalamic-pituitary-ovarian axis in PCOS (9-42).

This review summarizes the studies conducted over a given period that show the efficacy of these non-chemical and herbal substances in treating PCOS.

**Table 1:** Summary of the studies on the effect of common non-chemical and herbal medicine on PCOS


** Author, yr (ref)**	**Herbal compounds**	**Model/design**	**Duration/doses**	**Results (therapeutic effects on PCOS) or mechanism**
**Mahood ** ***et al.*** **, 2012 (10)**	Anise (*Pimpinella anisum*)	Rats/estradiol valerate induced PCOS	200 mg/kg, 400 mg/kg for 15 days	1. Decreased signs of PCOS in rats by effects on the histo-morphologies of the ovarian tissue 2. Ameliorated the hormonal profile of PCOS (FSH, LH, P4)
**Oyelami ** ***et al.*** **, 2012 (11)**	Sausage fruit (*Kigelia africana* [*Lam*] *Benth*)	Human trial (two PCOS patients)	1 tsp. of powder twice daily before food for almost two yr	1. Restored the menstrual flow / no side effects 2. Reduced acne but no noticeable effect on the hirsutism 3. Reduced size of the right ovary to normal due to the strong anti-inflammatory effect of the plant and presence of specific COX1 and COX2 inhibitors
**Wei ** ***et al.*** **, 2012 (12); Cai ** ***et al.*** **, 2012 (13); Wang, 2015 (14)**	Berberine (BBR), a major active component of the Chinese herbal medicines Rhizoma Coptidis, Cortex Phellodendri, and Cortex Berberidis	Human trial (89 women)	BBR at a dosage of 3x500 mg daily with three months duration	1. Reduced LDL, triglycerides, cholesterol, glucose, insulin and insulin resistance levels as well as increased HDL and SHBG 2. “BBR combined with Chinese prescription Cang Fu Dao Tan Tang reduced BMI, HOMA-IR, FIN-D2D, T, LH, and LH: FSH, LDL-C, and the effect on TG, LDL-C, and HDL-C” 3. “Mechanisms are still unclear”
**Karampoor ** ***et al.*** **, 2014 (15)**	Fennel (*Foeniculum vulgare*)	Rats/estradiol valerate induced PCOS	250, 500, 1000 mg/kg BW, for 4-10 days	Increased serum concentration of FSH and decreased LH and T
**Thakor ** ***et al.*** **, 2014 (16)**	Wild indigo (*Tephrosia purpurea*)	Rats/letrozole induced PCOS	200 mg/kg for at least three consecutive estrous cycles	1. “Normalized estrous cycle and steroidal hormonal levels (FSH, LH, T, E2)” 2. “Increased fertility in female rats, and reduced histopathological changes in ovary and endocrinological and biochemical changes due to hyperandrogenism”
**Fatima ** ***et al.*** **, 2015 (17)**	Flax seed (*Linum usitatissium*)	Open-label interventional study (32 women with PCOS)	Orally 15 gr flax seed powder for three months	1. “Reduced the ovarian volume and number of follicles and improved the menstrual cycles but did not alter the body weight, blood sugar or hirsutism” 2. “Positive effect on PCOS, due to the reduction in T, E2, LH and insulin levels which contributed to follicular maturation, and the anti-inflammatory actions to the reduction in ovarian volume”
**Kargar Jahromi ** ***et al.*** **, 2015 (18)**	Pomegranate (*Punica granatum*)	Rats/estradiol valerate induced PCOS	100 mg/kg, 200 mg/kg, 400 mg/kg, for 81 days	1. Reduced the effect of T hormone due to phenolic compounds present in the pomegranate extract 2. Reduced the complications associated with PCOS and improved changes of female sex hormones by reducing the concentration of E2, free T, and andrestandion hormones in PCOS
**Poornima ** ***et al.*** **, 2015 (19); Bhuvaneshwari ** ***et al.*** **, 2015 (20)**	Pergularia (*Pergularia daemia*)	Rats/testosterone propionate induced PCOS	1 ml fresh Pergularia leaves extract every day for 15 days	1. Ameliorated the essential hormones in the menstrual cycle: FSH, LH, E2, P4 and T, and thus their effect in infertility treatment 2. Reduced LDL, triglycerides, cholesterol and glucose levels in the serum, and helped manage obesity pattern in PCOS rats
**Swaroop ** ***et al.*** **, 2015 (21)**	Fenugreek seed (*Trigonella foenum-graecum*)	Open-label surveillance study (50 women with PCOS)	Two capsules of 500 mg/day for 90 days	1. Reduced both left and right ovarian volume and number of ovarian cysts but no significant adverse effects in serum ALT, BUN and CK 2. Increased LH and FSH levels and a small decrease in LH: FSH ratio 3. Effective in alleviating the symptoms of PCOS and demonstrated broad-spectrum safety and efficacy
**Zare ** ***et al.*** **, 2015 (22)**	Nettle (*Urtica dioica*)	Rats/letrozole induced PCOS	150 mg/kg, 250 mg/kg, 450 mg/kg, for 21 days	1. “Effective in decreasing some common symptoms of metabolic syndrome and type 2 diabetes in PCOS relating to its ability in adjusting the lipid profile and increasing the sensitivity to insulin, because of its flavonoid compounds” 2. “Increased insulin sensitivity, reduced hepatic necrosis and may reduce inflammation and improve metabolic symptoms in PCOS rats”
**Bergner ** ***et al.*** **, 2016 (23)**	Licorice (*Glycyrrhiza glabra*)	Human trial (seven men)	7 gr of a commercial preparation of licorice tablets for a wk	1. Decreased T 2. Inhibited conversion of androstenedione to T and might be useful for expressions of androgenization
**Demirel ** ***et al.*** **, 2016 (24)**	Hazelnut (*Corylus avellana*)	Rats/letrozole induced PCOS	2 ml for 45 days	1. “Ability to regulate gonadotropins, steroids, serum lipid parameters, and also it had antioxidant activity in PCOS, which could be attributed to the relatively high total phenol content of the extract” 2. Decreased leptin and glucose concentration
**Karimi Jashni ** ***et al.*** **, 2016 (25)**	Palm pollen (*Phoenix dactylifera*)	Rats/estradiol valerate induced PCOS	200 mg/kg, 400 mg/kg for 21 days	1. Reduced the number of cystic follicles, improved tissue symptoms and adjusted the levels of sex hormones in PCOS 2. “Increased the number of primary, antral and graaffian follicles as well as the corpus luteum”
**Kavitha ** ***et al.*** **, 2016 (26)**	Guggul (*Commiphora wightii*)	Rats/ dehydroepi- androsterone (DHEA)	100 mg/kg DHEA for 15 days	“Had a potential role in reducing DHEA-induced PCOS by reducing the morphological abnormalities of ovarian follicles and restoring hormonal levels to normal in adult rats”
**Radha ** ***et al.*** **, 2016 (27)**	Aloe vera (*Aloe barbadensis miller*)	Rats/letrozole induced PCOS	Treatment regime with Aloe vera gel	1. “Altered ovarian-placental steroid status by modulating the LH receptor, androgen receptor, aromatase and steroidogenic acute regulatory protein” 2. “Improved reproductive performance”
**Reddy ** ***et al.*** **, 2016 (28)**	Curcumin (found in *Curcuma longa* rhizomes)	Rats/letrozole induced PCOS	100 mg/kg, 200 mg/kg for 15 days	1. “Reduced fasting blood glucose levels and glycosylated hemoglobin levels in the serum” 2. “Restored the hormone and lipid profile, antioxidant and glycemic status, as well as ovarian morphology in PCOS animals because of its multiple pharmacological activities like estrogenic, antihyperlipidemic, antioxidant and hyperglycemic effects” 3. Increased the uterine weight so matched the normal rats
**Suhaimi ** ***et al.*** **, 2016 (29)**	Mistletoe fig (extract of leaves of *Ficus deltoidea*)	Rats/letrozole induced PCOS	25 mg/kg, 125 mg/kg, 250 mg/kg for 42 days	1. “Induced fewer cystic follicles with presence of a number of corpora lutea and various stages of developing follicles implying ovulation as compared to PCOS rats” 2. Decreased the ovarian wet weight and increased uterine wet weight of PCOS rats, and showed protective effects against ovaries and uterine in PCOS
**Zhou ** ***et al.*** **, 2016 (30)**	*Atractylodes macrocephala koids* (AMK)	Rats/testosterone propionate induced PCOS	0.9 gr/kg, 0.3 gr/kg, 0.1 gr/kg once a day for eight wk	1. “Improved the estrous cycle and reduced plasma levels of TT, androstenedione and FSH receptor expression” 2. “Increased aquaporin-9 in the rat's ovaries, and polar extract of AMK relieved PCOS and regulated FSH receptor and aquaporin-9 expression”
**Dawane ** ***et al.*** **, 2017 (31)**	Nishamalaki (a combination of *Curcuma longa* and *Emblica officinalis)*	Rats/letrozole induced PCOS	0.9 gr/kg for 56 days	1. Decreased body weight along with dyslipidaemia 2. Reduction in lipid profile, blood sugar and insulin, and effectively corrected all changes in PCOS
**Foroozandeh ** ***et al.*** **, 2017 (32)**	Ginger (*Zingiber officinale*)	Rats/letrozole induced PCOS	100, 200 and 300 mg/kg for 28 days	1. Increased FSH but no significant effect on the level of E2 and T hormones 2. Increased primary follicles, primary and secondary graviflower and yellow corpuscles, and decreased atritic follicles
**Rajan ** ***et al.*** **, 2017 (33)**	Soy isoflavone in soybean (*Glycine max*)	Rats/letrozole induced PCOS	50 mg/kg, 100 mg/kg for 14 days	1. “Led to significant recovery in the biochemical and clinical parameters” 2. Decreased body weight, percentage diestrous phase, T, 3B-HSD and 17B-HSD enzyme activity and oxidative stress 3. Well-developed antral follicles and normal granulose layer in rat ovary and aromatase activity
**Dou ** ***et al.*** **, 2018 (34)**	Cinnamon (a spice obtained from several tree species from the genus *Cinnamomum*)	Rats/dehydroepi- androsterone	10 mg/100 gr for 20 days	1. Improved insulin resistance due to inhibition of tyrosine phosphatase and improved insulin sensitivity, restored ciclicity, decreased IGF-1, increased IGFBP-1 and down-regulated T in PCOS rats 2. Hepatoprotective, antioxidant, anti-obesity, antihyperlipidemic and antidiabetic activities
**Jahan ** ***et al.*** **, 2018 (35)**	Quercetin (3,5,7,3 ' ,4 ' -pentahydroxyflavone)	Rats/letrozole induced PCOS	30 mg/kg for 21 days	1. “Potential to alleviate the hormonal and metabolic disturbances occurring in PCOS” 2. “Showed beneficial effects by decreasing body weight, ovarian diameter and cysts, and restoring healthy follicles, follicles' extra-glandular layers, and corpora lutea in contrast to the positive control” 3. “Regulated steroidogenesis by decreasing the levels of T and E2, and increasing P4 levels”
**Lee ** ***et al.*** **, 2018 (36)**	Welsh onion (*Allium fistulosum*)	Rats/letrozole induced PCOS	500 mg/kg for two wk	1. “Led to a low plasma LH: FSH ratio, high E2 levels, ovarian morphology, folliculogenesis-related gene expression” 2. Influenced aromatase production and enhanced E2 synthesis 3. “Restored the estrogenic feedback mechanism in the pituitary-ovary system”
**Tahmasebi ** ***et al.*** **, 2018 (37)**	*Calligonum polygonoides*	Rats/estradiol valerate induced PCOS	20 mg/kg for two months	1. As an antioxidant, decreased ovary cysts, oxidative stress and ROS, and eliminated free radicals in PCOS model 2. Improved in vitro fertilization rate and reduced weight
**Yang ** ***et al.*** **, 2018 (38)**	Brown alga(*Ecklonia cava*)	Rats/letrozole induced PCOS	*E. cava* extract per os (P.O.) for two wk	1. Restored the hormone levels, including T, E2, LH, FSH, and AMH 2. Restored the irregular ovarian cycles and inhibited the symptoms of PCOS
**Yang ** ***et al.*** **, 2018 (39)**	Licorice (*Glycyrrhiza glabra* L. and its varieties [1-7])	Rats/letrozole induced PCOS	300 mg/kg (prepared in 0.2% CMC) for two wk	1. Reduced the LH: FSH ratio and led to recovery of the FSH level 2. “Inhibited the symptoms of PCOS by regulating imbalanced hormonal levels and irregular ovarian follicles” 3. “Reversed histological changes, follicular cysts and antral follicles, and increased the thickness of the theca and granulosa layers observed in PCOS”
**Kakadia ** ***et al.*** **, 2019 (40)**	*Vitex negundo* (*Verbenaceae*)	Rats/letrozole induced PCOS	200 mg/kg, 400 mg/kg up to 66 days	1. “Restored the abnormality in serum sex steroid profile, LH: FSH ratio, steroidogenic enzymes, cardiovascular parameters, lipid profile and the glucose and estrous cycles” 2. “Exerted its protective effects by restoring parameters to the normal levels and leading to the disappearance of cysts in ovaries in PCOS rats due to its phyto-components”
**Miao ** ***et al.*** **, 2019 (41)**	Dodder (*Cuscuta*)	Rats/dehydroepi- androsterone, combined human chorionic gonadotropin	200 mg/kg, 100 mg/kg, 50 mg/kg for three wk	1. Decreased ovarian and uterine viscera indexes 2. Decreased LH: FSH ratio, serum P, PRL and INS levels, IGF-1 and TNF-α 3. Improved uterus and pancreas pathological changes (such as endometrial glandular hyperplasia, irregular or tubular arrangement)
**Ndeingang ** ***et al.*** **, 2019 (42)**	*Phyllanthus muellerianus* (*Euphorbiaceae*)	Rats/letrozole induced PCOS	30 mg/kg, 60 mg/kg, 120 mg/kg for seven or 14 days	1. “Alleviated the reproductive, biochemical, and structural alterations in PCOS rats characterized by the restoration of estrus cyclicity, the reduction of blood glucose levels and oxidative stress, as well as the improvement of the lipid profile and sex hormones” 2. Decreased cystic follicles, LH and T levels, but increased E2 concentration 3. “It was proposed that *P. muellerianus *acts by (a) modulating the pulsatile release of GnRH, LH, and FSH, (b) amplifying the aromatization of androgens into estrogens, and (c) stimulating estrogen production by adipocytes, responsible for the restoration of estrus cyclicity and ovulation induction”
**Shao ** ***et al.*** **, 2019 (9)**	Shaoyao-Gancao Decoction (SGD)	Rats/letrozole induced PCOS	12.5 gr/kg, 25 gr/kg, 50 gr/kg for 14 days	1. “Alleviated hyperandrogenism in PCOS rats as evidenced by reduced serum levels of T and increased E2 and FSH levels” 2. “Reduced the phosphorylation of NF-κB p65 and increased the expression of IκB” 3. “SGD could ameliorate hyperandrogenism in PCOS rats, and the potential mechanism may relate to the NF-κB pathway”
FSH: Follicle stimulating hormone, E2: Estradiol, T: Testosterone, LH: Luteinizing hormone, P4: Progesterone, PRL: Prolactin, AMH: Anti-Müllerian hormone, GnRH: Gonadotropin releasing hormone, LDL: Low density lipoprotein, LDL-C: Low density lipoprotein cholesterol, HDL: High density lipoprotein, HDL-C: High density lipoprotein cholesterol, SHBG: Sex hormone binding globulin, COX1: Cyclooxygenase 1, COX2: Cyclooxygenase 2, BMI: Body mass index, HOMA-IR: Homeostatic model assessment for insulin resistance, FIN-D2D: Finnish national diabetes prevention program, TG: Triglyceride, ALT: Alanine aminotransferase, BUN: Blood urea nitrogen, CK: Creatine kinase, ROS: Reactive oxygen species, P.O.: Per os, CMC: Carboxymethyl cellulose, PCOS: Polycystic ovary syndrome				

## 2. Materials and Methods

This review was carried out using relevant keywords in the electronic databases PubMed/MEDLINE, Web of Science, Scopus and Reaxys. Articles were limited to those published from January 2009 to December 2019 and we used the keywords “non-chemical treatment”, “herbal treatment”, “phytotherapy”, “polycystic ovary syndrome” and “PCOS”. We conducted searches to capture all animal studies and pre-clinical and clinical studies explaining the effects of herbal extracts on PCOS. In addition, we manually searched bibliographies of review articles. A total of 230 studies were found to be about the effects of herbal compositions on the treatment of PCOS. We excluded clinical and animal studies investigating herbal medicines with unrelated outcomes and finally 34 studies were selected.

## 3. Results

There have been numerous studies on the etiology of PCOS and its common and traditional herbal and non-chemical treatments (1-47). Each of these treatments has different effects. Most of them can partially improve metabolic and hormonal abnormalities. This can be an effective way for preventing and treating PCOS by affecting the factors involved in the disease. Some important experimental studies with significant results and human and clinical trials are described in table I. In most of these studies, the regulation and balance of the steroidogenic enzymes, sex steroid profile, LH: FSH ratio, lipid profile, glucose or insulin levels were reported, which aimed to improve and treat PCOS disease.

## 4. Discussion

This review lists laboratory, clinical, and animal studies which used phytotherapy to treat PCOS disease (9-42). The results demonstrated the importance and potential of herbal and non-chemical therapies to improve the hormonal status such as FSH, LH, their ratios and significant recovery in the biochemical and clinical parameters of PCOS. For example, in one study this was done through restoring the estrus cyclicity, decreasing cystic follicles, LH and testosterone levels, and increasing estradiol and FSH concentrations in PCOS rats treated with *Phyllanthus muellerianus *(*Euphorbiaceae*) (42). Another example was berberine, which in human trials, combined with Chinese herbs, showed positive effects on alleviating insulin resistance, and improving glycolipid metabolism and reproductive endocrine conditions (43).

Since there were different results from different herbal compounds in these studies, it is necessary to classify herbal compounds according to the hormonal and neurological factors and changes in gene expression level. A number of herbal compounds and substances can be given more attention as they control and influence hormones at the level of the central nervous system and its major axes in the reproductive system, including testosterone, estrogen, LH and FSH (9, 32, 38, 39, 41, 42). In some studies, the effect of neuropeptides such as from the galanin family have been evaluated in the treatment of PCOS disease (44, 45). PCOS patients have been shown to have significantly lower levels of nesfatin-1 (45) and also one study showed a small increase in galanin, but this was not significant compared to the control group (46). Recently, we demonstrated that galanin as a neuropeptide could ameliorate the metabolic and reproductive disturbances in a rat model of PCOS (47).

Determining the exact causes of the disease and its molecular basis can certainly play a key role in the treatment of PCOS using non-chemical and herbal ingredients and neuropeptides. Further pre-clinical, clinical and experimental studies are needed to show the effects of these non-chemical components for the management of PCOS.

## 5. Conclusion

This review demonstrated the variability and effects of phytotherapy and non-chemical treatments associated with PCOS disease. These findings may help future studies on the etiology and treatment of this syndrome, which is the most common cause of female infertility.

##  Conflict of Interest

The authors declare that there is no conflict of interest.
